# Endovascular repair of two acute type A aortic dissections with Gore thoracic branch endoprosthesis

**DOI:** 10.1016/j.jvscit.2025.101961

**Published:** 2025-08-28

**Authors:** Kinsing Ko, Guillaume Geuzebroek, Mark Dirven, Foeke Nauta, Sjoerd Jenniskens, Robin Heijmen

**Affiliations:** a Department of Cardiothoracic Surgery, Radboud University Medical Centre, Nijmegen, the Netherlands; b Department of Vascular Surgery, Radboud University Medical Centre, Nijmegen, the Netherlands; c Department of Medical Imaging, Radboud University Medical Centre, Nijmegen, the Netherlands

**Keywords:** TEVAR, TBE, Type A Aortic dissection

## Abstract

In this case report, we describe two patients with acute ascending aortic syndromes who were treated endovascular with a Gore TAG Thoracic Branch Endoprosthesis (W. L. Gore & Associates) in zone 0.

Thoracic endovascular aortic repair has become the standard of care in descending aortic pathologies. For the ascending aorta, open surgery remains the golden standard with excellent short and long-term outcomes.^1^ However, 8% to 28% of patients with acute type A aortic dissection are unfit for open surgery owing to advanced age and comorbidities.^2^^,^^3^ These patients could benefit from a far more less invasive, endovascular treatment. The literature includes case reports and small series describing off-label use of various devices.^4^^,^^5^ In this case report, we describe the off-label use of the new Gore TAG thoracic branch endoprosthesis (TBE) (W. L. Gore & Associates) in aortic zone 0 combined with proximal extension with the GORE TAG conformable thoracic stent graft (TAG) (W. L. Gore & Associates) for the treatment of two patients with an acute aortic syndrome of the ascending aorta (local type A aortic dissection). Both patients provided consent to publish their case.

## Case report

### Case 1

**Fig 1:**
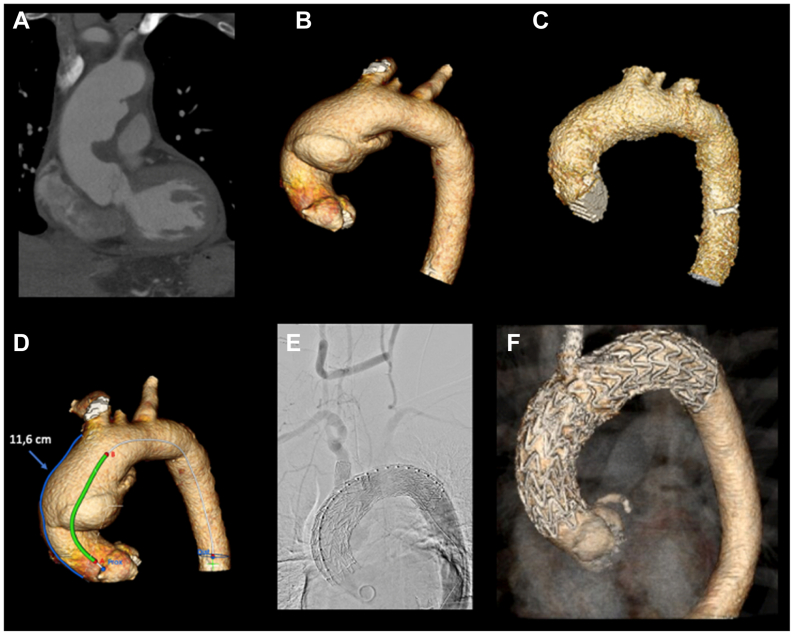
**(A)** Computed tomography (CT) scan showing a large intimal defect with a partially thrombosed false lumen of the ascending aorta. **(B)** Three-dimensional reconstruction in of the aortic dissection. **(C)** Three-dimensional reconstruction of the aorta from a CT scan that was made a few weeks earlier for the follow up of his malignancy. **(D)** Three-dimensional reconstruction and measurements from the sinotubular junction (STJ) up to the brachiocephalic trunk (BCT). **(E)** Intraoperative angiogram after stent deployment and patent right to left carotid bypass. **(F)** Three-dimensional reconstruction of the final result.

An 80-year-old man with a comprehensive oncological history (metastasized prostatic cancer to the liver and lungs and sigmoid carcinoma) was presented to one of our referring hospitals with acute chest pain. Computed tomography angiography (CTA) showed an acute, local type A aortic dissection (Fig 1, *A*-*F*) with a large intimal defect (ulcer-like projection) in the ascending aorta and a thrombosed false lumen from the ascending to the descending aorta. A few weeks earlier, a regular control CTA showed a normal aorta (Fig 1, *C*). Given his age and oncological medical history, patient was initially treated nonoperatively by means of pain and blood pressure management. After 10 days, a repeat CTA showed an increase of the intimal defect with false lumen growth and the referring cardiologist consulted us to reconsider our initial decision. Although his life expectancy was estimated to be more than 1 year, we still felt that open surgery was not opportune; thus, we developed with an endovascular strategy. This procedure was meticulously planned by our multidisciplinary complex aortic team, which consists of a cardiac surgeon, a vascular surgeon, and an interventional radiologist, and was discussed with the clinical specialist from W. L. Gore & Associates.

The patient was scheduled in the hybrid operating room the next day for a multidisciplinary, endovascular intervention. We started with a bypass from the right to the left common carotid artery. The left subclavian artery (LSA) was not revascularized, because both vertebral arteries had a similar diameter. A Gore TBE endograft was then implanted in zone 0 with the side branch into the brachiocephalic trunk (BCT). The TBE endograft covered the left common carotid artery and LSA and landed distally in zone 4. Last, the TBE endograft was extended proximally with a Gore TAG endograft just up to the sinotubular junction (STJ). All CTA measurements were performed using Endosize software (Therenva). We aimed for 0% to 10% oversizing.

### Case 2

**Fig 2:**
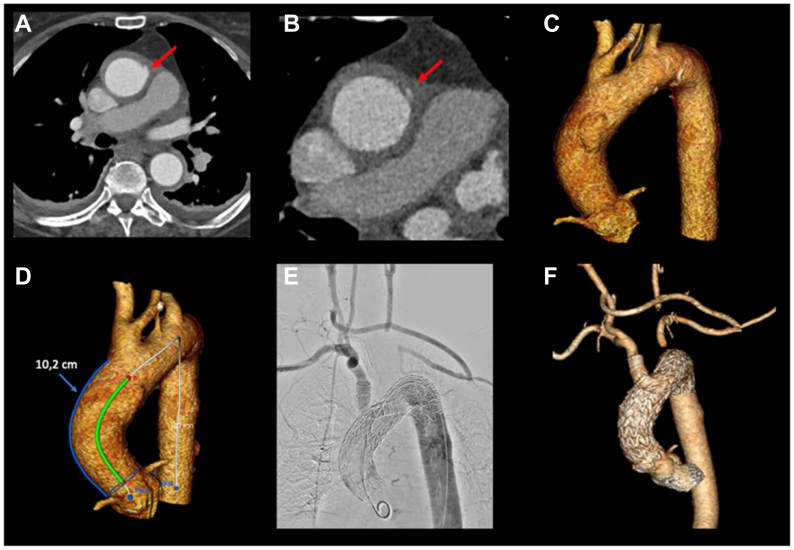
**(A** and **B)** Computed tomography scan showing a clear intimal defect in the ascending aorta with an intramural hematoma (IMH). **(C)** Three-dimensional reconstruction of the defect. **(D)** Three-dimensional reconstruction and measurement from the sinotubular junction (STJ) up to the brachiocephalic trunk (BCT). **(E)** Intraoperative angiogram after stent deployment and patent carotid-carotid-subclavian bypass. **(F)** Three-dimensional reconstruction of the final result, including the patent bypass.

A 77-year-old woman, with no relevant cardiovascular medical history, presented with chest pain to a referring hospital. CTA showed an acute, local, type A aortic dissection with an intimal defect (entry) in the ascending aorta and thrombosed false lumen (Fig 2, *A*-*F*). The aortic arch and descending aorta were unaffected. The patient was deemed unsuitable for open surgery by multiple cardiac centers because she refused blood products for religious reasons. A follow-up CTA showed an increase in the defect in the ascending aorta. After several days, our team was consulted to determine if we could develop an endovascular option. We agreed that open surgery was not feasible and explored whether a endovascular solution similar to the first case was possible.

Our surgical plan was almost identical to our previous case; however, because the vertebral artery originated from the arch, we revascularized the LSA. First a carotid-carotid-subclavian bypass was performed, followed by implantation of a Gore TBE endograft in zone 0 with the side branch into the BCT and proximal extension to the STJ with a Gore TAG endograft. All CTA measurements were again made with the Endosize software (Therenva). We aimed for 0% to 10% oversizing.

### Operative procedure

#### Case 1

The patient was scheduled in the hybrid operating room and was treated by our complex aortic team. First, a right-to-left carotid bypass was performed with the use of an 8-mm ringed vascular graft. The origin of the left common carotid artery was ligated. Access for the TBE endograft was obtained percutaneously in the right femoral artery (Gore Dryseal sheath 24F) and preclosure devices were deployed (Perclose ProGlide system, Abbott Vascular). The left femoral artery was used for an angiography catheter (7F sheath). Surgical cutdown of the right brachial artery was performed to accommodate the through-and-through wire for later implantation of the side branch into the BCT. The main device was then advanced over a stiff wire (Lunderquist, Cook Medical) and positioned in the ascending aorta. Blood pressure was temporary lowered by inferior caval vein occlusion with a Reliant balloon (Medtronic) without rapid pacing. After deployment of the TBE endograft in zone 0 (37 mm in diameter, 150 mm long, 12 mm portal diameter), which distally landed in zone 4, a side branch (15 mm) was deployed in the BCT. The Lunderquist wire was then changed for a Safari^2^ (Boston Scientific) stiff wire and positioned into the left ventricle. The Gore TAG (40 mm in diameter, 100 mm long) was then implanted just above the STJ connecting to the TBE endograft with a few centimeters of overlap. The outer curve length from the STJ until the BCT was 11.6 cm (Fig 1, *D*).

A perioperative angiogram showed no endoleak and patent coronary arteries. In the absence of any observed endoleak, postdeployment ballooning was not performed, except for the side branch, in accordance with the manufacturer's instructions for use. The systolic blood pressure in the left arm was 40 mm Hg lower than in the right arm. The patient experienced no limitations and had no complaints regarding his left arm. A postoperative CTA showed a small endoleak originating from the branch in the BCT. The patient was discharged 1 day after the procedure and scheduled for a second procedure to implant a 14 × 29 mm covered endograft (BeGraft, Bentley InnoMed) in the BCT 2 weeks later. The procedure was uncomplicated. A direct postoperative CTA revealed no endoleak (Fig 1, *E* and *F*). and the patient was discharged 1 day after surgery. His further recovery was uneventful.

#### Case 2

The procedure was similar to the first case, except that we also revascularized the LSA. The following surgical steps are similar to our first case: access the TBE endograft through the right groin with preclosure devices. A through-and-through wire was placed in the right brachial artery and right femoral artery, and a Gore TBE endograft was deployed in zone 0 (31 mm in diameter, 150 mm long, 12 mm portal diameter) and distally landed in zone 4, with its side branch (15-mm device diameter for a 12-mm portal) into the BCT and proximal extension with a Gore TAG endograft (34 mm in diameter, 100 mm long) in the ascending aorta. The outer curve length from the STJ until the BCT was 10.2 cm (Fig 2, *D*). An intraoperative angiogram and postoperative CTA showed no endoleaks or complications (Fig 2, *E* and *F*). As in our first patient, no postdeployment ballooning was performed, except for the side branch. The patient was discharged 6 days postoperatively and recovered uneventfully.

## Discussion

In this case report, we described the use of the TBE endograft combined with proximal extension with a Gore TAG endograft in two patients with acute aortic syndrome of the ascending aorta. It is a matter of debate whether this is truly a type A aortic dissection. The aortic layers are however clearly dissected. Especially in the first patient, we can appreciate that an earlier CTA did not show any sign of a penetrating ulcer or other atherosclerotic defects (Fig 1, *C*). The second case resembles an intramural hematoma (IMH) more closely; however, a large entry can be seen on the CTA (Fig 2, *A* and *B*).

Both patients were initially deemed unsuitable for (open) surgical repair. Probably owing to the local nature of the defect or dissection, they both survived the hyperacute phase and could be scheduled for a later complex endovascular repair. The postoperative course was uneventful for both patients and no neurological deficits developed. The TBE endograft by Gore became commercially available in Europe since 2024 and was approved in the United States in 2022. Several American groups have reported their results, of which most endografts were implanted in zone 2 and some in zone 0 or 1.^6^, ^7^, ^8^, ^9^, ^10^ For patients with an acute aortic syndrome, there are groups that have reported the use of the TBE endograft for the treatment of acute aortic pathology, mainly in zone 2, but also some for zone 0.^6^^,^^7^ Pitts et al^5^ reported the successful implantation of a TBE endograft in zone 0 with cervical debranching, but with proximal extension by use of an uncovered dissection stent (PETTIOAT technique). With the recent availability of the TBE endograft in Europe, we have shown that this technique (a TBE in zone 0 with proximal extension in patients with acute [local] type A aortic dissection) is feasible and reproducible in highly selected patients.

Both our patients had an intimal defect in the ascending aorta with a limited dissection of the aortic layers. This specific pathology likely resembles the more benign course of an IMH, instead of a full-blown DeBakey type 1 aortic dissection. In the most current European and American guidelines, early surgery for an IMH of the ascending aorta is recommended whenever there are high-risk features present.^1^ However, controversies remain; some investigators have reported comparable results of surgical vs nonsurgical treatment strategies.^11^ Although some IMHs can resolve spontaneously, progression can lead to rupture. In both our patients, there was progression of the disease; therefore, intervention was necessary.

Because open surgery remains the first choice of treatment in acute ascending aortic pathologies,^1^ endovascular intervention should only be considered for patients who are deemed unsuitable for open surgery. In both our cases, the patient was deemed unsuitable for open surgical repair and therefor an endovascular procedure was considered. After surviving the hyperacute phase, but with an increasing defect, we treated both patients with a TBE in zone 0 and proximal extended up to the STJ with a Gore TAG. Implanting a stent graft within a dissected aorta carries inherent risks, including the potential for stent-graft induced new entry tear or retrograde dissection. Consequently, it is preferable to deploy the proximal and distal ends of the stent graft within intact, unaffected segments of the aorta. In both our patients, this was feasible owing to the localized defect of the ascending aorta. Regarding the proximal landing zone, careful attention must be paid to avoid coverage of the coronary arteries. Given that the intervention was deliberately deferred, there was a (small) window of time for aortic remodeling and reinforcement of the aortic wall layers. Therefore, patient risk profiles and procedural risks must be interpreted within this context. Consequently, this technique requires further development and evaluation before it can be applied regularly to cases involving extensive dissection flaps in the setting of acute ascending aortic dissection. As described by Pitts et al,^5^ the use of an uncovered stent (PETTICOAT technique) might be one of the possibilities.

The endograft is available with two portal sizes: an 8-mm and a 12-mm portal for the side branch. After implantation of this endograft in the aortic arch, the length of the STJ to the portal of the TBE endograft is important for the feasibility of proximal extension with another stent graft. Most commercially available endografts have a minimum length of 10 cm, which is often longer than the ascending aorta. Therefore, the technique described in this case report is promising, but only applicable in patients with an elongated aorta. The availability of shorter stents will make this technique widely applicable in patients with ascending or aortic arch pathology, which will hopefully come with the results of ARISE.^12^

## Conclusions

In selected patients, hybrid endovascular treatment of an acute, local type A aortic dissection in carefully selected patients who are deemed unsuitable for open surgery is feasible with the use of a single branched thoracic device combined with proximal extension with a conventional endograft. This technique is currently particularly suitable for patients with a longer ascending aorta. Further development of shorter stents will make this technique more widely applicable.

## Funding

None.

## Disclosures

None.
